# Mpox multi-antigen mRNA vaccine candidates by a simplified manufacturing strategy afford efficient protection against lethal orthopoxvirus challenge

**DOI:** 10.1080/22221751.2023.2204151

**Published:** 2023-05-08

**Authors:** Jiawei Zeng, Yao Li, Linrui Jiang, Ling Luo, Yue Wang, Hao Wang, Xiaonan Han, Jian Zhao, Guanglei Gu, Min Fang, Qingrui Huang, Jinghua Yan

**Affiliations:** a CAS Key Laboratory of Pathogenic Microbiology and Immunology, Institute of Microbiology, Chinese Academy of Sciences, Beijing, People’s Republic of China; b University of Chinese Academy of Sciences, Beijing, People’s Republic of China; c College of Life Sciences, Anhui Agricultural University, Hefei, People’s Republic of China; d College of Life Sceinces, Henan University, Kaifeng, People’s Republic of China; e Changping Laboratory, Beijing, People’s Republic of China

**Keywords:** Mpox, mRNA vaccine, multi-antigen, VACV challenge, simplified manufacturing

## Abstract

Current unprecedented mpox outbreaks in non-endemic regions represent a global public health concern. Although two live-attenuated vaccinia virus (VACV)-based vaccines have been urgently approved for people at high risk for mpox, a safer and more effective vaccine that can be available for the general public is desperately needed. By utilizing a simplified manufacturing strategy of mixing DNA plasmids before transcription, we developed two multi-antigen mRNA vaccine candidates, which encode four (M1, A29, B6, A35, termed as Rmix4) or six (M1, H3, A29, E8, B6, A35, termed as Rmix6) mpox virus antigens. We demonstrated that those mpox multi-antigen mRNA vaccine candidates elicited similar potent cross-neutralizing immune responses against VACV, and compared to Rmix4, Rmix6 elicited significantly stronger cellular immune responses. Moreover, immunization with both vaccine candidates protected mice from the lethal VACV challenge. Investigation of B-cell receptor (BCR) repertoire elicited by mpox individual antigen demonstrated that the M1 antigen efficiently induced neutralizing antibody responses, and all neutralizing antibodies among the top 20 frequent antibodies appeared to target the same conformational epitope as 7D11, revealing potential vulnerability to viral immune evasion. Our findings suggest that Rmix4 and Rmix6 from a simplified manufacturing process are promising candidates to combat mpox.

## Introduction

Since May 2022, the number of mpox infection cases, previously endemic in Africa, has increased dramatically in non-endemic countries [1]. The World Health Organization (WHO) has declared that the global mpox outbreak represents a public health emergency of international concern [1]. Mpox is a zoonotic viral disease caused by the mpox virus, a large, enveloped double-stranded DNA orthopoxvirus (OPV) in the *Orthopoxvirus* genus of the *Poxviridae* family [1]. The *Orthopoxvirus* genus also includes highly pathogenic variola virus (VARV) and vaccinia virus (VACV) [2]. Immunization with an orthopoxvirus vaccine provides cross-protective immunity against other orthopoxvirus [3]. Therefore, two live-attenuated VACV-based vaccines (ACAM2000 and JYNNEOS), initially developed in the smallpox eradication programme, have been approved by the US Food and Drug Administration to prevent mpox [4]. However, these vaccines are associated with several serious adverse events (AEs), including myocarditis and pericarditis, especially in certain individual groups such as people with eczema and pregnant women [3,4]. So far, they are approved only for preexposure prophylaxis among persons at high risk for such exposures rather than general public use [4]. Therefore, new vaccines that are safer, more efficacious, and more specific for mpox are urgently needed to combat the potentially increasing mpox epidemic in the context of worldwide decreasing herd immunity against smallpox.

Orthopoxviruses have a large and complex proteome with over 200 proteins [3,5]. During infection, mature virions (MV) and enveloped virions (EV) exist, which contain approximately 25 and 6 surface proteins, respectively [5]. Mice immunization with individual VACV protein (A33, B5, or L1) or with any combination of two proteins was protective, but the combination of all three proteins was the most effective [6,7]. In non-human primate models, although both a VACV trivalent (A33, B5, and L1) and tetravalent (trivalent plus A27) protein-based adjuvanted vaccine provided protection from lethal mpox virus challenge, the tetravalent vaccine clearly outperformed the trivalent vaccine for viral loads and clinical outcomes [8]. Similarly, a DNA vaccine consisting of four VACV genes (A33, B5, L1, and A27) protected monkeys from severe disease following a lethal mpox virus challenge [9]. Human antibodies induced by VACV infection recognize diverse MV and EV surface proteins, the majority of neutralizing antibodies targeted one of six antigens (MV proteins A27, L1, H3, D8, and EV proteins B5 and A33) and exhibited cross-neutralization for several orthopoxvirus species [5]. Including multiple antigens seems necessary to elicit an optimal immunological response during mpox vaccine development. mRNA vaccine takes advantage of safety, versatility, rapid development, and induction of potent B and T cell responses as evidenced by two SARS-CoV-2 mRNA-based vaccines [10]. Generally, each mRNA encoding an antigen needs to be synthesized and encapsulated separately to manufacture a multi-antigen mRNA vaccine. An optimized strategy of manufacturing a multi-antigen mRNA vaccine in a single-process manner would have a huge advantage in cost and manufacturing convenience. Here we developed two mRNA vaccine candidates against mpox that encode mpox four (M1, A29, B6, A35) or six (M1, H3, A29, E8, B6, and A35) antigens and demonstrated that both multi-antigen vaccine candidates afforded significant protection against VACV challenge in a mouse model. Moreover, a simplified strategy of preparing a multi-antigen mRNA vaccine in a single process was shown to be able to produce an immunogenicity-competent mRNA vaccine.

## Materials and methods

### Ethics statement

This study was approved and conducted strictly following the recommendations stated in the Guidelines for the Care and Use of Laboratory Animals issued by the Ethics Committee of the Institute of Microbiology, Chinese Academy of Sciences.

### Cells, viruses, and animals

HEK293T cells (ATCC, CRL-3216) and BSC-1 cells (ATCC, 3168) were cultured at 37°C in Dulbecco's Modified Eagle medium (DMEM) with 10% fetal bovine serum (FBS). Initial stock of VACV Western Reserve was obtained from Dr. Luis Sigal (Thomas Jefferson University). Specific-pathogen-free (SPF) 6-8-week-old female mice BALB/c and C57BL/6 were purchased from Beijing Vital River Animal Technology Co., Ltd (licensed by Charles River). Body weight of each mouse ranged from 18 g to 20 g. We used a “Completely Randomised” method to randomly allocate mice into intervention or placebo groups [11]. For example, there were 18 mice that needed to be randomly allocated into three treatments (A, B, and C) with a sample size of six. Briefly, the mice were first numbered from 1 to 18 using ear tags. Six “A”s, six “B”s and six “C”s were entered into column one and 18 random numbers were put in column two using the command “ = rand()”, and pulling down on the small box on the lower right of the cell. Columns one and two were then marked and sorted on column two using “data, sort”. The row numbers represent individual mice identification numbers. All mice used in this study were housed and bred in temperature-, humidity- and light- cycle-controlled SPF mouse facilities in IMCAS (20 ± 2°C; 50 ± 10%; light, 7:00–19:00; dark, 19:00–7:00).

### Protein expression and purification

The optimized sequences of mpox virus H3, M1, A29, A35, B6, E8, and VACV H3, L1, A27, D8, B5, A33 fused with N-terminal native signal peptides and C-terminal 8× His tag respectively, then cloned into the pCAGGS expression vector (Addgene). The plasmids were transiently transfected into HEK293T cells. After 5 days, the supernatant was collected and purified by a HisTrap HP 5 ml column (GE Healthcare). Each sample was purified via size-exclusion chromatography with a Superdex 75 column (GE Healthcare) in PBS. To prepare antibodies against the antigens of mpox, the variable region links to the sequence encoding the constant region of mouse IgG2a, resulting in a full-length H chain and L chain plasmid. MAbs expressed by co-transfection of HEK293T cells. The antibodies were isolated by Hitrap Protein A 5 mL column (GE Healthcare) and purified by Superdex 200 column (GE Healthcare) in PBS.

### mRNA production and purification

mRNA was synthesized by T7 RNA polymerase using linearized plasmids as a template as previously described [10]. The linearized plasmids encode codon-optimized mpox virus H3, M1, A29, A35, B6, and E8 proteins and contain antigen protein open reading frame flanked by the 5’-UTR, 3’-UTR, and 104-nt polyA tails. Antigen protein coding sequence fused with N-terminal native signal peptides and C-terminal 8× His tag, respectively. After production, mRNA was precipitated with LiCl overnight at −20°C, centrifuged at 15000 ×g for 50 min at 4°C to pellet, purified with 70% ethanol, centrifuged at 15000 ×g for 1 min at 4°C, and dissolved in RNase-free water. The purified mRNA was stored frozen at −80°C until use.

### mRNA transfection and flow cytometry

HEK293T cells were seeded in 12 well plates (3 × 10^5^ cells/well) for transfection. mRNA (1 μg) was transfected into HEK293T cells with TransITmRNA (Mirus Bio) according to the Manufacturer's instructions. After 24–48 h, the HEK293T cells were collected and resuspended for the next detection. To detect protein expression, the cells were fixed and permeabilized using a BD Cytoperm fixation/permeabilization solution (BD Biosciences) and then stained respectively with each antigen monoclonal antibody expression supernatant in wash buffer for 30 min on ice escape antigen M1. HEK293T cells that expressed M1 were stained with His antibody PE (Miltenyi Biotec). Whereafter, cells were incubated with Alexa Fluor® 488 Goat Anti-Mouse IgG (H + L) (YEASEN) for 30 min on ice in the dark. Data were acquired on a BD FACSCanto flow cytometer (BD Biosciences) and analyzed with FlowJo 10.6.2.

### Lipid-nanoparticle encapsulation of mRNA

The mRNA was encapsulated with lipid nanoparticles as previously described [10]. Briefly, the mRNA was diluted with an aqueous solution of mRNA at pH = 4.0 to a final concentration of 100 μg /ml. Then the solution of mRNA was rapidly mixed with lab-prepared lipids dissolved using Nano Assemblr Ignite (PrecisionNanoSystems). Lab-prepared lipids dissolved contained an ionizable cationic lipid, phosphatidylcholine, cholesterol, and PEG-lipid, and the ratio of which was 50:10:38.5:1.5 mol/mol. The mRNA-LNP formulations were diluted with Phosphate Buffered Saline (PBS) and concentrated by AmiconUltra Centrifugal Filter Unit (Millipore) to desired concentrations. The concentration and encapsulation rate of mRNA were measured by Quant-it RiboGreen RNA Assay Kit (Invitrogen, #R11490). The formulations were passed through a 0.22 μm filter and stored at 4°C. Lmix vaccine, an equal mass of multiple mpox virus antigen mRNA, was mixed after being encapsulated by lipid nanoparticles. Rmix vaccine, an equal mass of multiple mpox virus antigen line linearized plasmids were mixed, and then the mixture was transcribed into mRNA that formed a mixture of antigens mRNA.

### Quantitative real-time PCR

To detect Rmix4/6 proportion of different antigen components and stability between batches by HiScript II One Step qRT-PCR SYBR Green Kit (Vazyme, China) following the Manufacturer's protocol. Each antigen plasmid was diluted at a 10-fold ratio to produce a standard curve (R^2^ > 0.99, 90%<amplification efficiency < 105%) to estimate the corresponding copies of mRNA. Since the mRNA mass of each Lmix component is equal, it can be used as a calibration sample. Calculate the relative value of each antigen for Rmix (test) compared to Lmix (calibration), then the relative value of each antigen / the relative value of all antigens acquired the ratio of the different antigen components in Rmix (test). Primer sequences are as follows, forward primer: H3 5’-accaagcgagacctttcctaac-3’, reverse primer: 5’-ttgtcgtcgtcgcggatg-3’; A29 5’-acagagttcttcagcaccaagg-3’, reverse primer: 5’-gtcgtttcttttacagcacttctcg-3’; E8 5’-ctctgatgccagactgaaaaccc-3’, reverse primer: 5’-tagtcatcttccttgccccagt-3’; M1 5’-aagtgcgacatcgaaatcggc-3’, reverse primer: 5’-ggtgaacatggcgggcac-3’; A35 5’-agaatcagcatggtgatctccctg-3’, reverse primer: 5’-cttgtggtcgtactgtgtggtg-3’; B6 5’-ggaccctaacgctgtgtgt-3’, reverse primer: 5’-ccattcttctcttcgcatctgaagt-3’.

### Mice immunization

BALB/c mice (n = 6) or C57BL/6 mice (n = 7) were immunized through the intramuscular (i.m.) route with mpox antigen-encoding mRNA vaccine candidates or a placebo at day 0 and 14. The blood samples were collected at days 13 or 28. The sera isolated were used for determination of antigen-specific antibody or neutralization antibody titres. Immunized mice were anesthetized using isoflurane, executed by cervical dislocation [12], and spleens were taken for investigation of antigen-specific cellular immune response in intracellular cytokine staining (ICS) and enzyme-linked immunospot (ELISPOT) assays or for construction of antigen-specific B cells library.

### Enzyme-linked immunosorbent assay (ELISA)

Antigen-specific antibody titres were determined by ELISA [13]. The antigen for each mpox or VACV was overnight-coated with 50 mM carbonate–bicarbonate buffer (pH 9.6) at 4°C on Corning® 96-well clear polystyrene microplates at 200 ng per well. The microplates were blocked with 5% skim milk for 1 h at 37°C and discarded. Add 100 μL of twice serially diluted mouse serum and incubate at 37°C for 1 h. The microplates were washed three times using PBST, and an HRP-labelled anti-mouse Fc secondary antibody (Yeasen) was added. Then, 50μL of 3,3’,5,5'-Tetramethylbenzidine (Beyotime Biotechnology) was used as the substrate, and the reaction was stopped with 50 μL of 2 M sulfuric acid. Absorbance was measured at 450 nm using a microplate reader (PerkinElmer). The endpoint antibody titre was the highest dilution at which the serum produced a light absorption value (OD450) 2.1 times higher than the background value. To detect total antibody titres for mpox or VACV, 100 ng of each antigen was added to each well for coating, and the next step was performed as described above.

### ICS assay

ICS assay was performed to detect antigen-specific CD4+ T cell and CD8+ T cell immune responses [14]. The splenocytes from each immunized C57BL/6 mice were cultured with RPMI 1640 containing 10% FBS in 96-well flat-bottom plates (2 × 10^6^ cells/well). Meanwhile, 0.1 MOI of VACV was diluted in RPMI 1640 containing 10% FBS. The cultured splenocytes were stimulated with 0.1 MOI of VACV (100 μl/well) for 6 h and then incubated with Golgiplug (BD Biosciences) for an additional 12 h at 37°C, 5% CO2 incubator. The cells were washed with PBS containing 1% BSA to stain with anti-CD3, anti-CD4, and anti-CD8 surface markers (Biolegend) for 30 min at 4°C in the dark. After that, the cells were fixed and permeabilized using a BD Cytoperm fixation/permeabilization solution (BD Biosciences) and then stained with a cocktail of antibodies: IL-2 (Biolegend), IL-4 (Biolegend), INF-γ (Biolegend), TNF-α(Biolegend). Data were acquired on a BD FACSAria III flow cytometer (BD Biosciences) and analyzed with FlowJo 10.6.2.

### ELISPOT assay

IFN-γ and IL-2-based ELISPOT assays were performed to detect antigen-specific T lymphocyte responses [10]. Spleens of C57BL/6 mice were removed after one week of booster immunization, and splenocytes were isolated. 96-well flat-bottom plates were pre-coated with 10 μg/ml of anti-mouse IFN-γ Ab or anti-mouse IL-2 Ab (BD Biosciences, USA) and stored at 4°C overnight. RPMI 1640 containing 10% FBS was added with 200 μl/well and incubated for 2 h at room temperature to block the wells. Then, 0.1 MOI of VACV was diluted in RPMI 1640 containing 10% FBS and added 100 μl/well to ELISPOT plate microwells. Meanwhile, the cell suspension was prepared at 1 × 10^7^ cells/ml, and 100 μl/well of cell suspension was added to each well which was incubated in a 37°C, 5% CO_2_ incubator. After 24 h of incubation, the cells were removed, and the plates were processed in turn with biotinylated IFN-γ or IL-2 detection antibody (BD Biosciences, USA), streptavidin-HRP conjugate (BD Biosciences, USA), and AEC substrate (BD Biosciences, USA). After waiting 15 min for visible spots, the plates were washed clean with deionized water and dried. The number of spots was counted, and the ELISPOT plate microwells were photographed using an automatic ELISPOT reader and image analysis software (Cellular Technology Ltd.).

### VACV neutralization assays

Neutralizing activity of sera was determined using VACV in a plaque reduction neutralization (PRNT) assay [5]. Sera from mice vaccinated with the indicated vaccines were serially diluted 2-fold with 2.5% FBS RPMI 1640 Medium (from 1:5), dilutions of sera 50 μl were mixed with 50 μl VACV (100 PFU) incubated at 37°C for 1 h. Monolayers of BSC-1 (African green monkey kidney) cells in 12-well plates were then infected with the incubated Serum-VACV mixture for 1 h at 37°C. After infection, a semisolid 2% methylcellulose of Earle's basal minimal essential medium overlay was added to the wells, and cells were incubated at 37°C under 5% CO_2_ for 48 h. After 2 days incubator, monolayers BSC-1 were fixed with 4% paraformaldehyde at room temperature for 30 min, then stained with 1% crystal violet solution. Last, monolayer cells were rinsed with water, and the plaques were counted. The percent neutralization was calculated relative to the number of plaques in the absence of sera.

### VACV comet tail inhibition assays

For the comet reduction assay [15], confluent monolayers of BSC-1 cells in six-well cell culture plates were infected with VACV at 50 PFU/well in RPMI medium containing 2% FBS for 1 h at 37°C. The Viral Solution was then discarded and washed twice with PBS. Mice serum was diluted in RPMI medium containing 2% FBS at a 1:40 ratio, and 1.5 ml was added to each well. The plates were incubated at 37°C for 48 h. Cells were fixed with 4% paraformaldehyde after 72 h incubation and stained with crystal violet to observe comet tail formation.

### VACV mouse challenges

On two weeks after the second immunization, the BALB/c mice (n = 6) were intraperitoneally injected with an indicated lethal dose of VACV (5 × 10^6^ PFU) diluted in 100 µl PBS. The weight of each mouse was measured immediately before the challenge, and the day of the challenge was considered to be day 0. Each mouse's body weight and survival were measured daily for 14 days after the challenge. Mice that reached endpoint criteria and/or had 30% weight loss were humanely euthanized.

### Isolation and construction of antigen-specific B cells library

Cell suspension derived from the spleen of immunized mice, then positively selected using EasySep™CD19 microbeads (STEMCELL) according to the Manufacturer's manual. After acquiring CD19-positive cells, His labelled protein as bait incubated for 30 min, then staining 7AAD-Percp, CD93-APC, and His-PE. Gated as 7AAD-, CD93-, His+, to obtain antigen-specific BGC, sorted by BD FACSAria II flow cytometer (BD Biosciences). The target cells were collected and sent to Beijing Emei Tongde biotechnology company for B cells library construction and sequencing. Last, we obtained information on the V(D)J sequences generated by the 10 × Chromium platform.

### Biolayer interferometry assay (BLI)

Expression of antibodies and real-time binding was performed by biolayer interferometry using an Octet RED96 biosensor (Pall ForteBio) [16]. Briefly, the AMC biosensor from ForteBio was used to capture antibodies from cell cultures within 300 s to detection of expression. For binding experiments, a biosensor was used to capture the antibody for 300 s, then moved into a final concentration of 100 nM antigen protein and bound for 300 s, and next moved into PBST buffer to dissociate the antibody from the antigen protein. To identify antibody competition against L1 antibody 7D11, the M1 protein is first immobilized on the NTA sensor, then moves to the first antibody and binds for 600 s until saturation, followed by the second antibody. The experiments generally consist of M1-antibody1-antibody 2 as group 1, M1-PBST-antibody 2 as group 2, and M1-antibody 1–0 as a control group, while biosensor in the second is saturated the signal intensity Group 1 rising <20% of the group 2 implies complete competition.

### Statistical analyses and reproducibility

Statistical analysis of data from three biologically independent replicate experiments was performed using GraphPad Prism version 8.0 software (GraphPad, San Diego, CA, USA). Error bars displayed on graphs represent the mean ± SEM. Statistical significance was analyzed using unpaired Student's t-test for two groups. *P < 0.05, ** *P* < 0.01, *** *P* < 0.001, and **** *P* < 0.0001 were considered significant.

## Results

### Construction and characterization of mpox multi-antigen mRNA vaccine candidates

**Figure 1.**
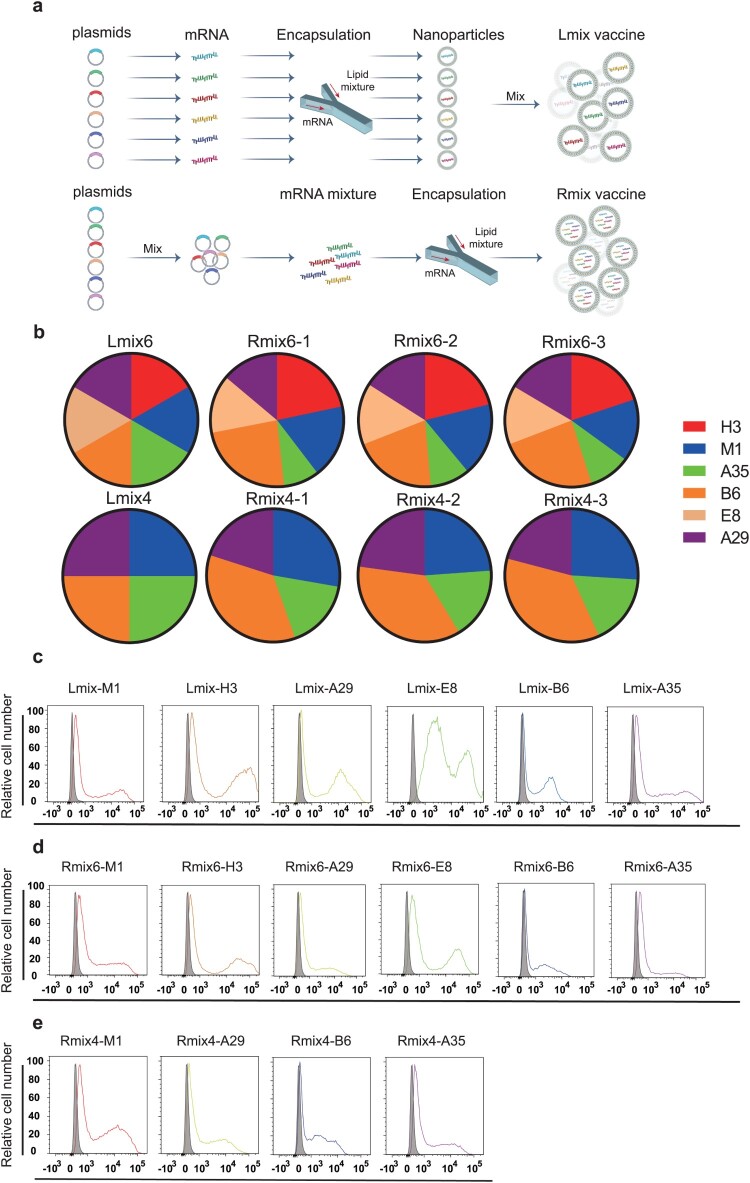
Construction and characterizations of mpox multi-antigen mRNA vaccine candidates. a, Schematic diagram of formulation procedure for multi-antigen mRNA vaccine candidates. b, Proportions of each antigen mRNA in three batches of Rmix4/6 and Lmix4/6. c-e, Representative images of antigen mRNA expression. Each antigen-encoding mRNA, Rmix4 or Rmix6, was transfected into HEK293T cells. HEK293T cells that expressed M1 were stained with His antibody PE. The other groups of HEK293T cells were stained with each antigen-specific monoclonal antibody and analyzed by flow cytometry. The bindings between antigens and their monoclonal antibody are shown in different colours. Grey shade indicates negative binding.

We designed two multi-antigen mRNA vaccine candidates, termed as mix4 (M1, A29, B6, and A35) and mix6 (M1, A29, H3, E8, B6, and A35). Individual mRNA was separately transcribed and further encapsulated with lipids to form lipid nanoparticles (LNPs) [10]. Then different LNPs were mixed in equal mRNA mass into the vaccine candidate formulations, named Lmix4/6 (Figure 1a). To facilitate vaccine manufacturing, we optimized the formulation procedure by mixing plasmids in equal mass and transcribing them into mRNA as a whole, which was then encapsulated with lipids to formulate the vaccine candidates, named Rmix4/6 (Figure 1a). The mRNA encapsulation efficiency is 96.4% for B6, 96.7% for A35, 96% for A29, 96.2% for M1, 96.7% for H3, 97.2% for E8, 96.1% for Rmix4, and 96.7% for Rmix6. We did a quantitative real-time PCR to detect proportions of different antigen components in Rmix4/6 and stability between batches. The results showed no significant difference between the three batches of Rmix4/6 in the proportions of different antigen mRNAs (Figure 1b and Fig. S1). When compared to Lmix, the ratios of different antigens in Rmix4/6 slightly changed. The B6 and A35 antigens showed the highest and lowest ratios in both Rmix4 and Rmix6, respectively (Figure 1b and Fig. S1). Those indicated that different antigen gene sequences may influence mRNA transcription efficiency. To verify protein expression profiles of different antigen-encoding mRNAs, we transfected mRNA into HEK293T cells. We analyzed them by flow cytometry using each antigen-specific monoclonal antibody. When compared to the control, each mRNA used in Lmix4/6 formulations showed robust protein expression, and all mRNA components in Rmix4/6 formulations expressed all the desired antigen proteins (Figure 1c-e).

### Immunogenicity of mpox multi-antigen mRNA vaccine candidates

**Figure 2.**
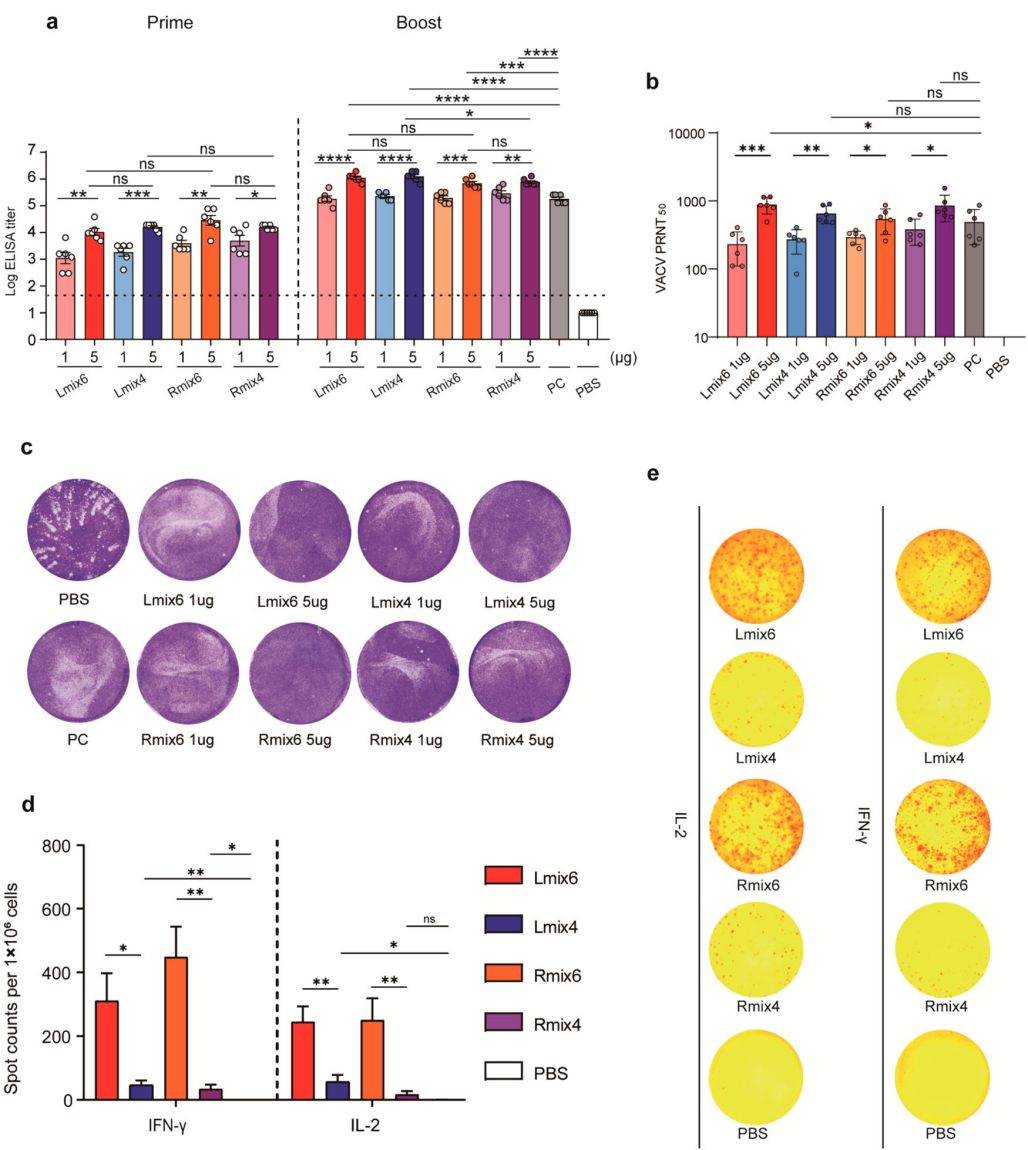
Immunogenicity of multi-antigen mRNA vaccine candidates in mice. Female BALB/c mice (n = 6) were immunized intramuscularly (i.m.) with two doses (1 µg or 5 µg/dose) of multi-antigen mRNA vaccine candidates or with a placebo. a, all six used antigens of mpox (B6, A35, A29, E8, M1, and H3) were mixed at a mass ratio of 1:1:1:1:1:1 and coated in 96-well plates to test antigens-specific antibody titres by ELISA. b, neutralizing activity of sera against MV was determined by plaque reduction neutralization (PRNT) assay against VACV infection, and 50% plaque reduction neutralization (PRNT_50_) was calculated. c, neutralizing activity of sera against EV was evaluated by comet inhibition assay. d,e, Cellular immune response of female C57BL/6 mice (n = 7) after vaccination with two doses of vaccine (5 µg/dose) or with placebo was measured by ELISPOT assay. IFN-γ and IL-2 secretion of splenocytes after stimulation with VACV were detected to evaluate cellular immune responses. Data are group means ± SEM. *P*-values were determined with t-test (ns, *P* > 0.05; *, *P* < 0.05; **, *P* < 0.01; ***, *P* < 0.001; ****, *P* < 0.0001).

The immune responses induced by mpox multi-antigen mRNA vaccine candidates were analyzed in BALB/c mice. Groups of female BLAB/c mice (n = 6) were immunized intramuscularly (i.m.) with two doses (1 µg or 5 µg) of Lmix4/6 or Rmix4/6 vaccine candidates at an interval of 14 days. PBS was immunized as a negative control. Serum samples were collected on days 13 and 28 following vaccination. Convalescent sera were collected from mice on day 14 following infection with 5 × 10^5^ PFU VACV and was used as a positive control (PC). Mpox purified antigens (B6, A35, A29, E8, M1, and H3) were mixed in equal mass and coated in 96-well plates to detect antigens-specific antibody titres by enzyme-linked immunosorbent assay (ELISA). All mice immunized with mpox vaccine candidates were positive seroconversion after primary immunization, and the antibody titres were significantly enhanced after the second immunization (Figure 2a). There was no significant difference in antibody titres between Lmix4/6 and Rmix4/6 at the same dose (Figure 2a).

Moreover, the antibody titres induced by the vaccine candidates were dose-dependent. The antibody titres of the high-dose (5 µg) groups were significantly higher than those of the low-dose (1 µg) groups (Figure 2a). Individual antigen-specific antibody titres were also detected by ELISA. As expected, immunization with Rmix6 and Lmix6 elicited high antibody titres targeting all six antigens. In contrast, the Rmix4 and Lmix4 elicited M1-, A29-, B6-, and A35-specific antibody responses (Fig. S2). As all six mpox antigens shared more than 91% sequence identity with the corresponding antigens from VACV and VARV (Fig. S3), we evaluated whether antibodies induced by mpox multi-antigen vaccine candidates could cross-react with VACV. We found that consistent with the high sequence identities, high VACV antigens-specific antibody titres were detected in all mpox-vaccinated sera (Fig. S4 and 5).

To assess virus-neutralizing antibody levels induced by mpox vaccine candidates, the serum 50% plaque reduction titres (PRNT_50_) against the VACV MV virions were measured. The PRNT_50_ titres of the high-dose (5 µg) groups were significantly higher than the low-dose (1 µg) groups, which were 850 vs. 201 for Lmix6, 629 vs. 246 for Lmix4, 502 vs. 287 for Rmix6, and 803 vs. 357 for Rmix4 (Figure 2b). Sera from mRNA-vaccinated mice also inhibited VACV transmission in the comet inhibition assay, indicating that mpox vaccine-elicited sera inhibited the formation of VACV EV particles.

Enzyme-linked immunospot (ELISPOT) and intracellular cytokine staining (ICS) assays were performed to assess the cellular immune response elicited by mpox multi-antigen vaccine candidates. C57BL/6 mice (n = 7) were vaccinated with two doses of 5 µg of Lmix4/6 or Rmix4/6, and one group was vaccinated with PBS as a negative control. The splenocytes were isolated 7 days following the second immunization and stimulated with 0.1 multiplicity of infection (MOI) of VACV to re-activate the antigen-specific T cells. The ELISPOT results showed that both Lmix6 and Rmix6 vaccine candidates induced significantly higher numbers of IFN-γ- and IL-2-secreting T cells than those of Lmix4 and Rmix4 (Figure 2 d,e). The ICS assays also demonstrated that Lmix6 and Rmix6 induced the highest ratios of IFN-γ secreting CD4+ T cells and CD8+ T cells when compared to Lmix4, Rmix4, and PBS (Fig. S6). These results indicated the additional two antigens (H3 and E8) in Lmix6 or Rmix6 vaccine candidates could promote potent T cell immune responses.

### Protective efficacy of mpox multi-antigen mRNA vaccine candidates against fatal orthopoxvirus challenge

**Figure 3.**
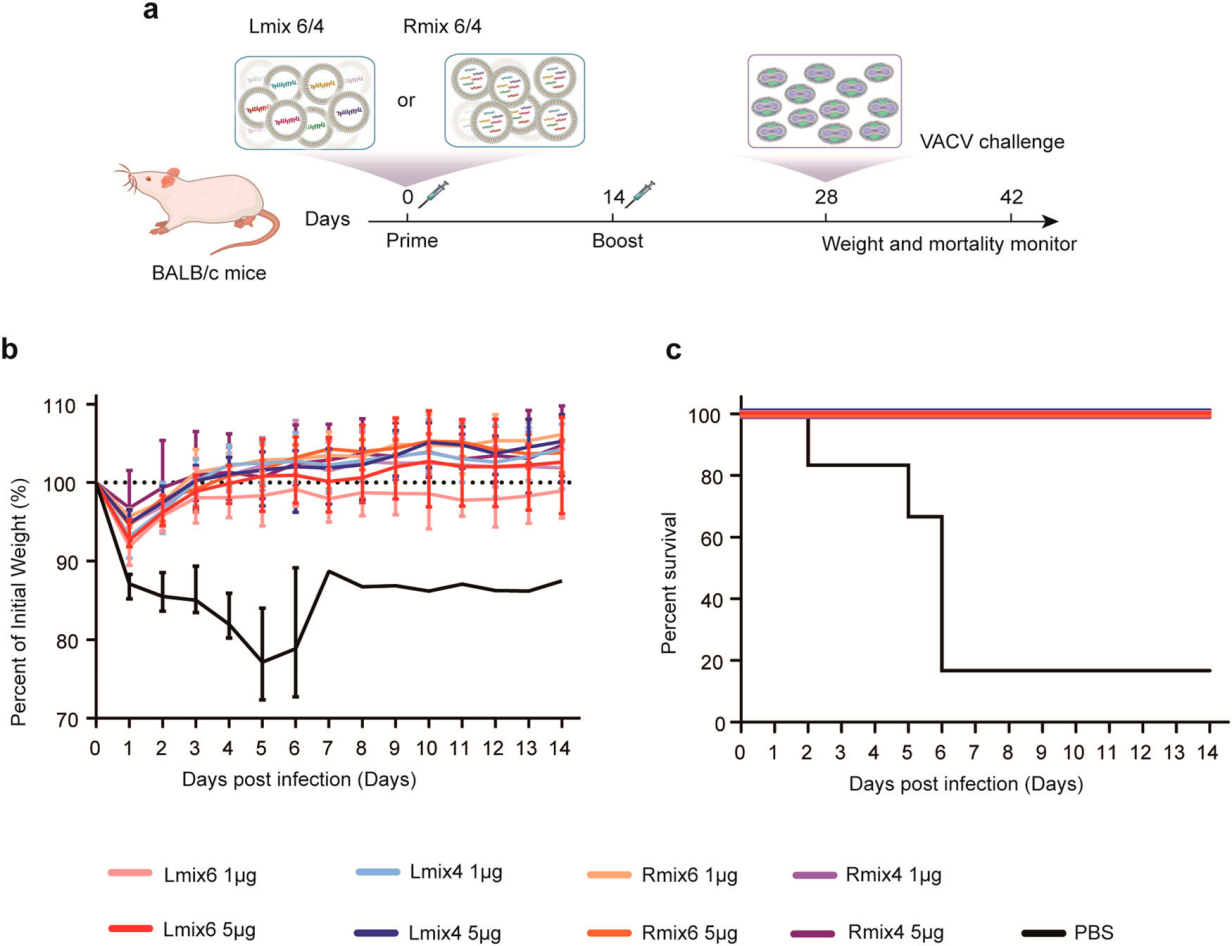
Cross-protective efficacy of mpox multi-antigen mRNA vaccine candidates against fatal VACV challenge in mice. a, Schedule of immunization and viral infection in mice. Female BALB/c mice (n = 6) were immunized i.m. with two doses (1 µg or 5 µg/dose) of multi-antigen mRNA vaccine candidates, 14 days apart. On day 14 following the second immunization, mice were infected intraperitoneally (i.p.) with 5 × 10^6^ PFU of VACV, and changes in body weight (b) and survival (c) of mice were monitored for 14 days.

To evaluate the protective efficacy of mpox multi-antigen mRNA vaccine candidates against fatal orthopoxvirus infection in mice, groups of BALB/c mice (n = 6) were immunized with two doses of 1 µg or 5 µg of the Lmix6, Lmix4, Rmix6, or Rmix4 vaccine candidates, and one group was immunized with PBS as a negative control. At 14 days post the booster vaccination, all immunized mice were challenged with a fatal dose (5 × 10^6^ PFU) of VACV via intraperitoneal (i.p.) route (Figure 3a). We recorded the body weight for each mouse daily after infection for 14 days. We found that 83.3% (5/6) of the mice immunized with PBS died, and the surviving one mouse also lost about 10% of initial weight. In contrast, all mRNA vaccine-immunized mice survived and maintained a stable weight at the pre-infection level (Figure 3b, c), demonstrating that the mpox multi-antigen mRNA vaccine candidates afforded efficient cross-protection against fatal VACV infections.

### BCR repertoires elicited by mpox individual antigen vaccinations in mice

**Figure 4.**
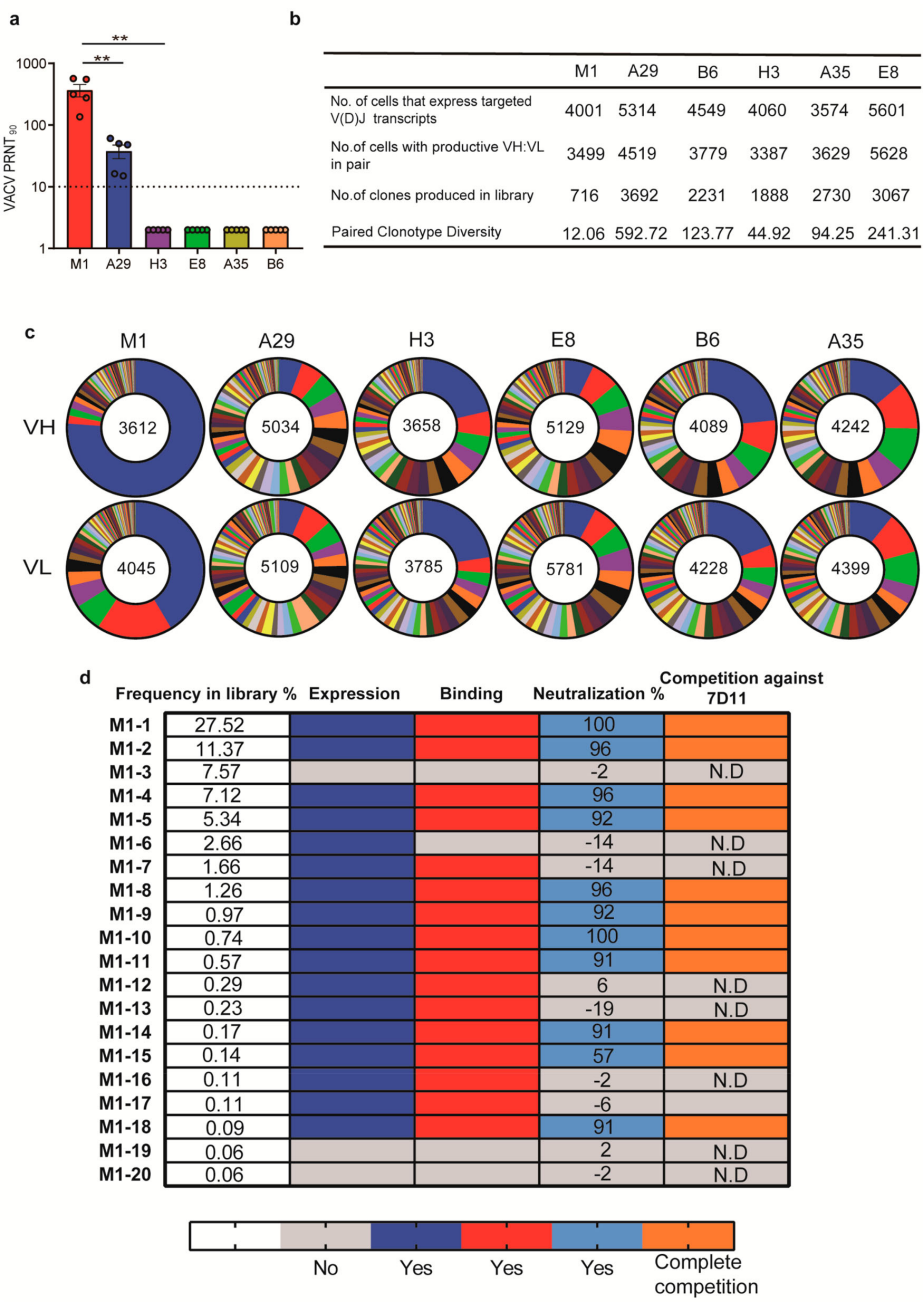
BCR repertoires elicited by mpox individual antigen vaccinations in mice. Female BALB/c mice (n = 5) were immunized i.m. with two doses of each antigen-encoding mRNA vaccine candidate or with a placebo, 14 days apart. Serum samples were collected on day 28. a, neutralizing activity of sera induced by each antigen-encoding mRNA vaccination was detected by plaque reduction neutralization assay against VACV. 90% plaque reduction neutralization (PRNT_90_) was calculated. b, Summary of the statistics for the scBCR-seq. c, Pie charts show clonal expansion in B6, A35, A29, E8, M1, and H3 specific BGC cells for IGVH (upper panel) or IGVL (bottom panel). Coloured slices are proportional to the number of clonal relatives. d, Antibody characteristics of the top 20 frequent mAbs in antibody repertoire elicited by M1 mRNA vaccine vaccinations. The dark blue, red, light blue, and orange indicate the antibody characteristics of expression, binding, neutralization, and complete competition against reference 7D11, respectively. Antibody expression, binding, and competition characteristics were assayed by Octet. Neutralizing activity of each mAb-expressed cell supernatant was determined by plaque reduction neutralization (PRNT) assay. Data are group means ± SEM. *P*-values were determined with t-test (ns, *P* > 0.05; *, *P* < 0.05; **, *P* < 0.01; ***, *P* < 0.001; ****, *P* < 0.0001).

To study B-cell receptor (BCR) repertoires elicited by each mpox individual antigen, groups of BALB/c mice (n = 5) were immunized with two doses of B6, A35, A29, E8, M1, or H3 mRNA vaccine candidates at a four-week internal, and PBS was used as a negative control. Serum samples obtained two weeks following booster vaccination were subjected to a neutralization assay to determine the 90% plaque reduction titres (PRNT_90_) against VACV. We found that the sera of the M1 antigen group exhibited the highest PRNT_90_ titres, followed by the A29 group. In contrast, all sera from the other antigen or PBS groups failed to reach a neutralization potency of PRNT_90_ even at a serum dilution of 1:5. Those indicated that M1 was superior in eliciting neutralizing antibody response to the other five antigens (Figure 4a). At two weeks following booster vaccination, antigen-specific memory B cells from mouse spleen were sorted by flow cytometry and then placed into the 10× Genomics for high-throughput single-cell V(D)J sequencing [16]. As a result, 3000∼6000 cells per antigen group that expressed targeted V(D)J transcripts were obtained and further sequenced. Of note, paired clonotype diversity of M1 was only 12.06. In contrast, the diversity of A29, B6, H3, A35, and E8 was 592.72, 123.77, 44.92, 94.25, and 241.31, respectively (Figure 4b). Consistent with a low clonotype diversity, the antibody germline usage in M1 BCR repertoire exhibited a strong enrichment. The ratios of the most frequent VH and VL germline were as high as 75% and 40%, respectively, implying that there is possibly one dominant super-epitope in the M1 antigen (Figure 4c). Given the excellent neutralizing potency of M1-elicited sera, we further characterize its BCR repertoire by recovering the most frequent 20 monoclonal antibodies (mAb) sequences, termed M1-1 to M1-20. The frequency of synthesized mAbs in the library was 68.05% (Figure 4d). Notably, among 20 mAbs, all 11 neutralizing mAbs showed a complete competition with 7D11, and either D35N or D35Y single substitution of M1 abrogates all neutralizing mAb bindings, indicating that all the most frequent 11 neutralizing mAbs in the BCR repertoire elicited by M1 targeted the same dominant epitope and are prone to neutralization escape. (Figure 4d, Fig. S7 and S8).

## Discussion

In this study, we developed mpox 4- and 6-antigen mRNA vaccine candidates by using an optimized strategy of manufacturing multi-antigen mRNA vaccine in one single process and demonstrated that both Rmix4 and Rmix6 vaccine candidates afforded efficient cross-protection against VACV fatal challenge in an animal model. Of note, the Rmix6 vaccine candidate elicited a much stronger T cell response than the Rmix4 one, indicating that Rmix6 may have a greater clinical application potential. One main limitation of our study is that due to limited access to the mpox virus, we applied an alternative VACV virus to rapidly assess the immunogenicity and protection efficacy of our mpox mRNA vaccine candidates. Although, in theory, an mpox vaccine candidate can generally afford a higher or at least similar efficacy against mpox itself than that against its close VACV virus, accurate vaccine effectiveness needs to be investigated by further mpox challenge studies.

Our study demonstrated that all recovered high-frequent neutralizing mAbs elicited by the mpox M1 antigen could potently neutralize VACV in a complement-independent manner and appeared to target the same conformational epitope as 7D11, which is surface exposed and distal to the transmembrane domain [17]. Consistently, Kaever et al. previously discovered that all anti-L1 (the corresponding protein in VACV to M1 in mpox) neutralizing mAbs recognized the same conformational epitope that was known to be the target of previously described 7D11 and other anti-L1 mAbs such as 2D5 and 10F5, and the epitope included Asp35 as a key residue [17,18]. In addition, VACV with either D35N or D35Y substitution can completely escape neutralization by those anti-L1 mAbs [18]. Therefore, despite the ability to elicit a potent neutralizing antibody immune response, an mpox vaccine candidate based on one single M1 antigen is likely vulnerable to neutralization escape.

mRNA vaccines can be classified into two groups-prophylactic vaccine and therapeutic vaccine [19]. After injection, mRNA vaccine is endocytosed by host immune or non-immune cells such as muscular cells and subsequently translated into protein that is displayed to T and B cells by MHC class I or II molecule [20]. Moreover, mRNA molecule can activate innate immune pathways, including TLRs, and lead to enhancement of B-cell and T-cell immune responses [21]. The innate immunogenicity of mRNA is an advantage for vaccine application. COVID-19 has accelerated the development of prophylactic mRNA vaccines including BNT162b2 and mRNA-1273. In the BNT162b2 and mRNA-1273 phase 3 studies, more than 80% of participants reported local adverse events, mainly pain [21–23]. The systemic events were mainly headache, temperature elevation, and myalgia [21–23]. Therapeutic mRNA vaccines are usually used for cancer therapy and expected to induce a strong cytotoxic CD8+ T cell response [21]. There are several cancer-therapeutic mRNA vaccines on clinical trials like BNT111 (NCT04526899), CV9202 (NCT03164772), and mRNA-4157 (NCT03313778) [21]. Clinical results showed that fatigue, injection-site soreness, colitis, and myalgia are common side effects of cancer mRNA vaccine [21]. However, long-term adverse effects of mRNA vaccine remain to be determined.

For multi-antigen mRNA vaccine development, although encapsulating all mRNAs at once is more effective, encapsulating each mRNA encoding an antigen separately is a usual choice. The separate LNP preparation allows to conveniently verify immunogenicity of each mRNA vaccine component [24]. Arevalo et al. developed a universal influenza mRNA vaccine against all known influenza virus subtypes by mixing 20 HA-encoding mRNA-LNPs together [24]. In addition, for a combined vaccine, when one vaccine was firstly developed and licensed to market, and another vaccine was subsequently developed, manufacturing each mRNA-LNP separately is usually necessary [25]. To our best knowledge, our study is the first report that the simplified strategy of mixing DNA templates in the initial step can produce a multi-antigen mRNA vaccine with similar immunogenicity and protective efficacy as the one from the traditional strenuous manufacturing process. In theory, our optimized manufacturing strategy also allows convenient ratio adjustments of different mRNA components in the vaccine by adjusting the corresponding plasmid ratios in the DNA template mixture. Moreover, the simplified strategy is also suitable for manufacturing multivalent or combined mRNA vaccines such as a respiratory virus combination vaccine against SARS-CoV-2, influenza, and respiratory syncytial virus.

## Author contributions

J.Y. and Q.H. designed the study; J.Z, Y.L., L.J., L.L., Y.W., H.W., X.H., J.Z., and G.G. conducted all assays. Q.H., J.Z, Y.L., and L.J. analyzed and interpreted the data. Q.H. wrote the manuscript. Q.H., J.Y., and M.F. discussed and edited manuscript.

## Supplementary Material

Supplemental Material

## Data Availability

The authors declare that the data supporting the findings of this study are available within this paper or are available from the corresponding author upon reasonable request.
