# Supplementary material for: Mpox multi-antigen mRNA vaccine candidates by a simplified manufacturing strategy afford efficient protection against lethal orthopoxvirus challenge
Source: Emerg Microbes Infect. 2023 May 8;12(1):2204151. doi: 10.1080/22221751.2023.2204151 (PMC10167873; doi:10.1080/22221751.2023.2204151)
Supplement: Supplemental Material [file TEMI_A_2204151_SM8479.docx]

**Supplementary Information File**

Mpox multi-antigen mRNA vaccine candidates by a simplified manufacturing strategy afford efficient protection against lethal orthopoxvirus challenge

Jiawei Zeng^a,b^†, Yao Li ^a^†, Linrui Jiang ^a,b^†, Ling Luo^b^, Yue Wang ^a,b^, Hao Wang ^a,b^, Xiaonan Han^a^, Jian Zhao^d^, Guanglei Gu ^a,b^, Min Fang^a,b^*, Qingrui Huang ^e^*, and Jinghua Yan ^a,b,e^*

^a^CAS Key Laboratory of Pathogenic Microbiology and Immunology, Institute of Microbiology, Chinese Academy of Sciences, Beijing 100101, China.

^b^University of Chinese Academy of Sciences, Beijing 101408, China.

^c^College of Life Sciences, Anhui Agricultural University, Hefei 230036, China

^d^College of Life Sceinces, Henan University, Kaifeng 475001, China

^e^Changping Laboratory, Beijing 102206, China

*Correspondence to: yanjh@im.ac.cn (Jinghua Yan); huangqr@cpl.ac.cn (Qingrui Huang); fangm@im.ac.cn (Min Fang).

†These authors contributed equally to this work.

**
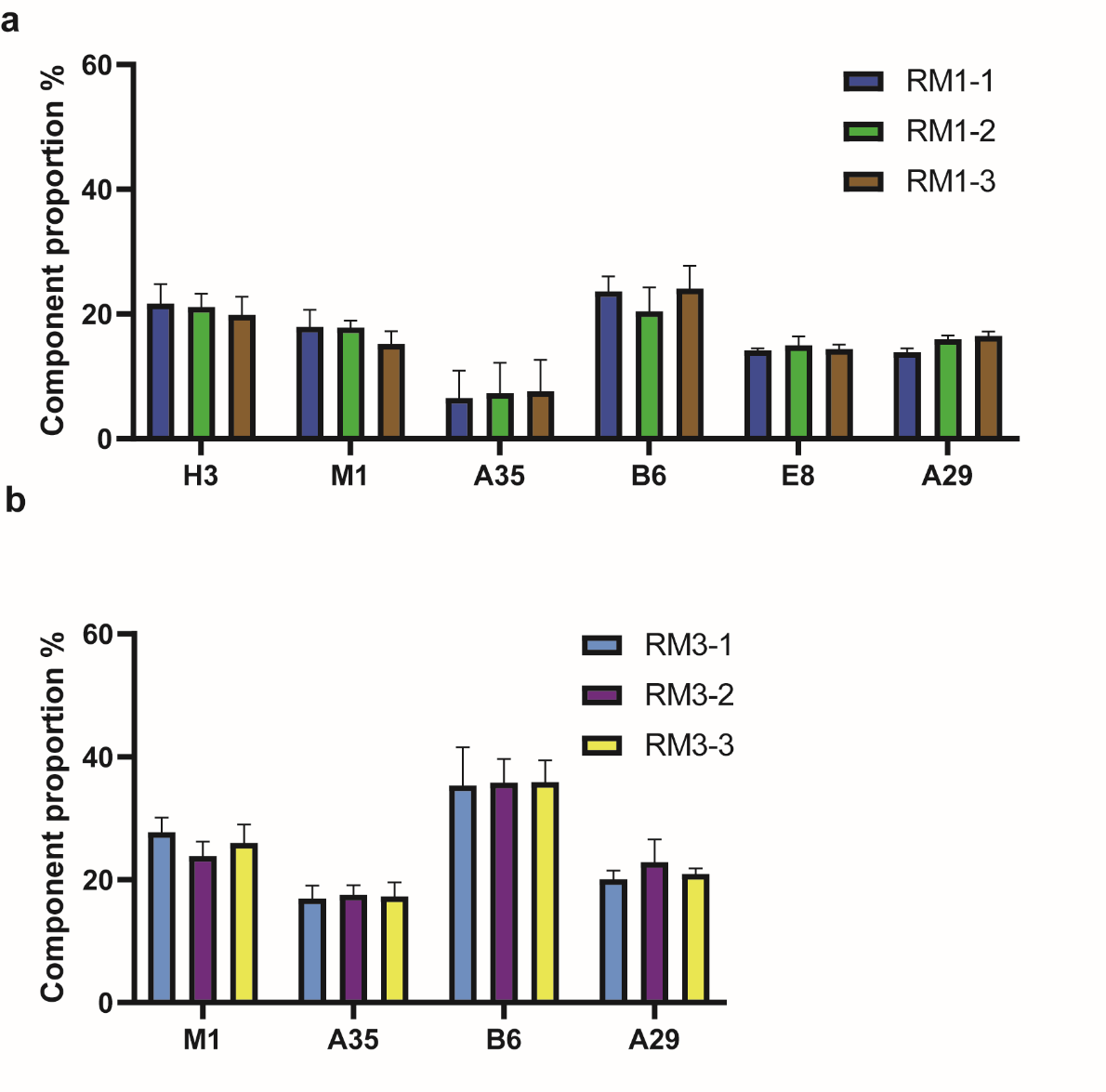
**

**Supplementary Figure 1. Proportions of different antigen mRNAs in three batches of Rmix6 and Rmix4 specimens.** Proportions of different antigen-encoding mRNAs in three batches of Rmix6 (**a**) and Rmix4 (**b**).


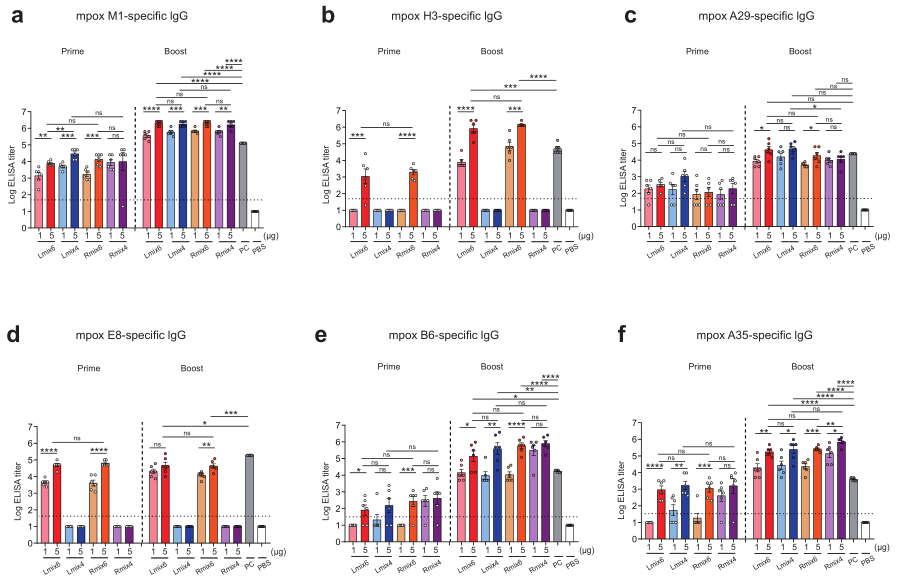


**Supplementary Figure 2. Each antigen-specific antibody titer of mouse sera induced by mpox multi-antigen vaccine candidates.** ELISA analyses of binding of immunized sera on captured mpox M1 **(a)**, mpox H3 **(b)**, mpox A29 **(c)**, mpox E8 **(d)**, mpox B6 **(e)**, and mpox A35 **(f)**. Data are group means ± SEM. P-values were determined with t-test (ns, *P* > 0.05; *, *P* < 0.05; **, *P* < 0.01; ***, *P* < 0.001; ****, *P* < 0.0001).


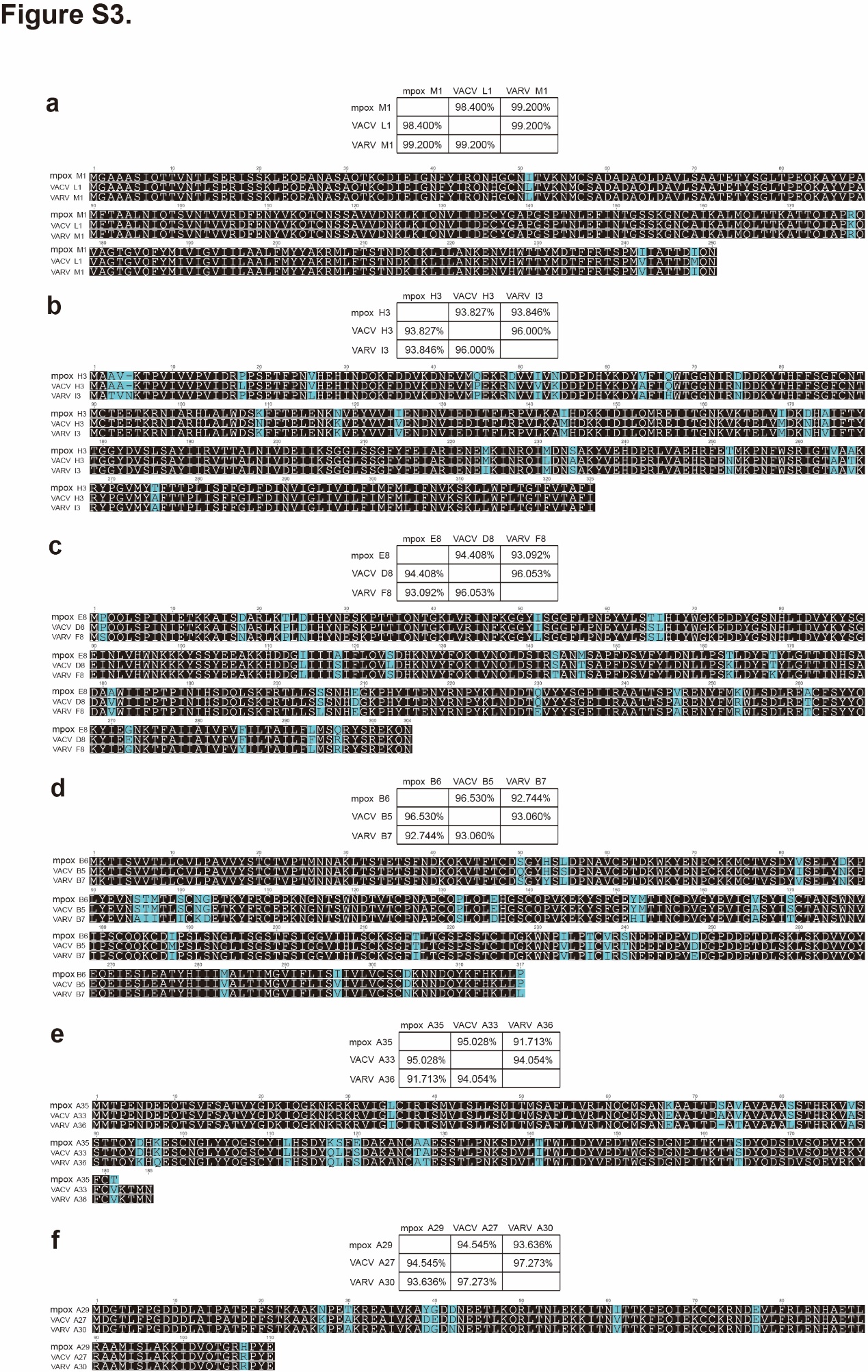


**Supplementary Figure 3. Homology alignment analysis of the antigenic sequences of mpox, VACV, and VARV. a-f,** Mpox antigen sequences were referred to publicly available reference genomes: ON563414.3 for the West African mpox strain, NC_006998.1 for the VACV Western Reserve strain, and NC_001611.1 for the VARV.


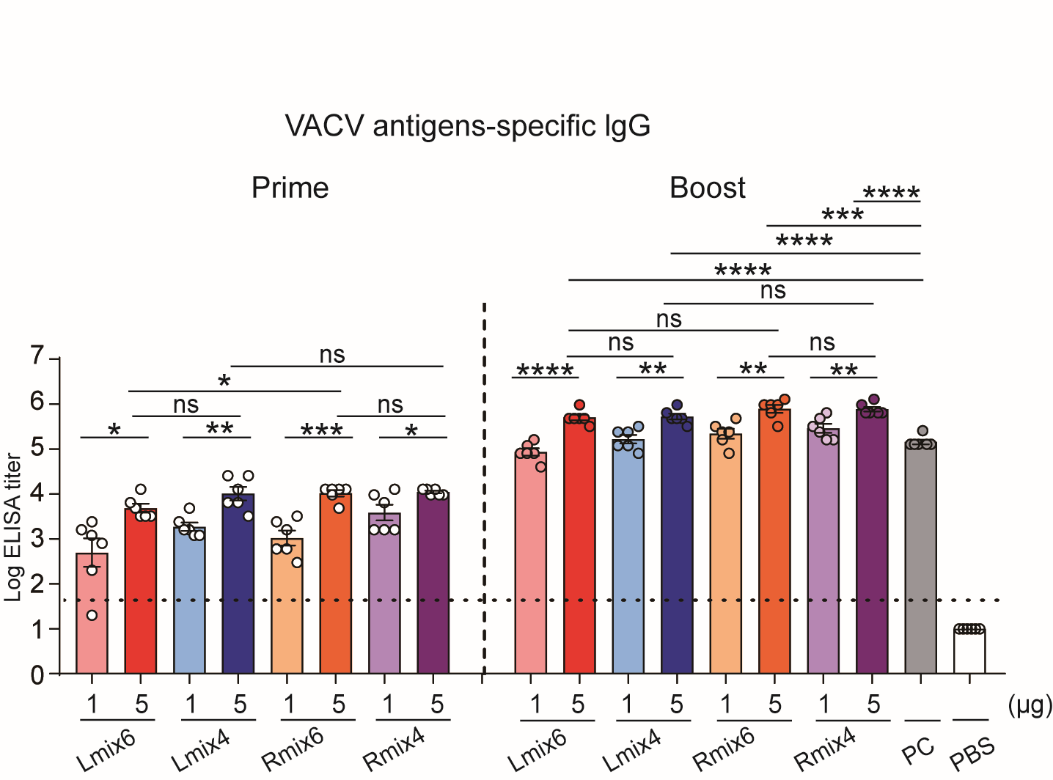


**Supplementary Figure 4. Cross-binding antibody titers of mpox-vaccinated sera against VACV antigen mixture.** **a,** all six VACV antigens (B5, A33, A27, D8, L1, and H3) were mixed at a mass ratio of 1:1:1:1:1:1 and then plated in a 96-well plate to determine cross-binding antibody titers in mpox-vaccinated sera by ELISA. Data are group means ± SEM. P-values were determined with t-test (ns, *P* > 0.05; *, *P* < 0.05; **, *P* < 0.01; ***, *P* < 0.001; ****, *P* < 0.0001).


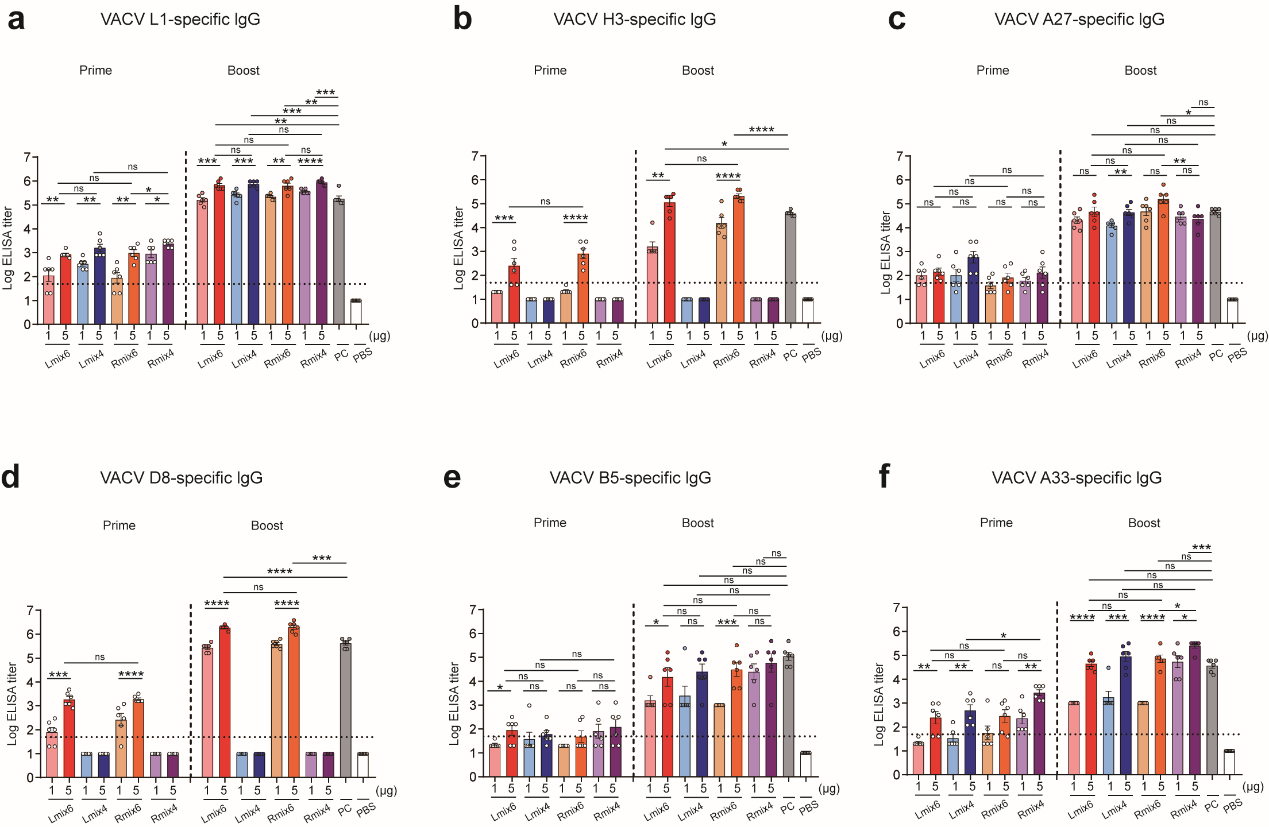


**Supplementary Figure 5. Cross-binding antibody titers of mpox-vaccinated sera against individual VACV antigen.** ELISA analyses of cross binding of mpox-immunized sera on captured VACV L1 **(a)**, VACV H3 **(b)**, VCAV A27 **(c)**, VACV D8 **(d)**, VACV B5 **(e)**, and VACV A33 **(f)**. Data are group means ± SEM. P-values were determined with t-test (ns, *P* > 0.05; *, *P* < 0.05; **, *P* < 0.01; ***, *P* < 0.001; ****, *P* < 0.0001).


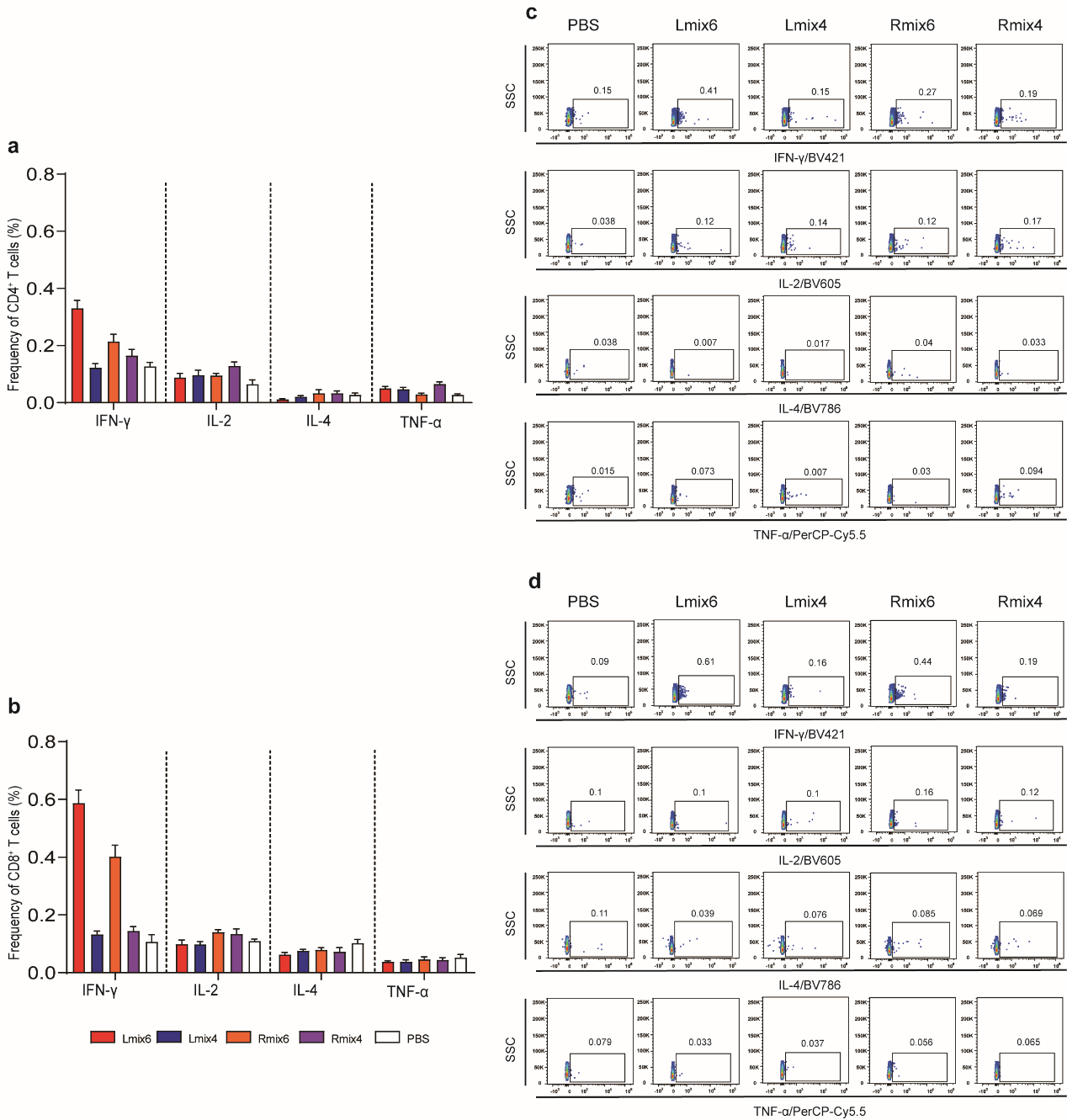


**Supplementary Figure 6. Determination of the cellular immune response induced by multi-antigen mRNA vaccine candidates in mice.** ICS assay was conducted to quantify the ratios of cytokine-positive T cells. Splenocytes from immunized C57BL/6 mice (n = 6) were stained with anti-CD3, anti-CD4, anti-CD8, anti-IFNγ, -IL-2, -IL-4, and -TNFα fluorescent antibodies. Proportions of cytokine-secreting CD4^+^ **(a,c)** and CD8^+^ **(b,d)** T cells were determined by flow cytometry. Data were acquired on a BD FACSAria III flow cytometer (BD Biosciences) and analyzed with FlowJo 10.6.2.

**
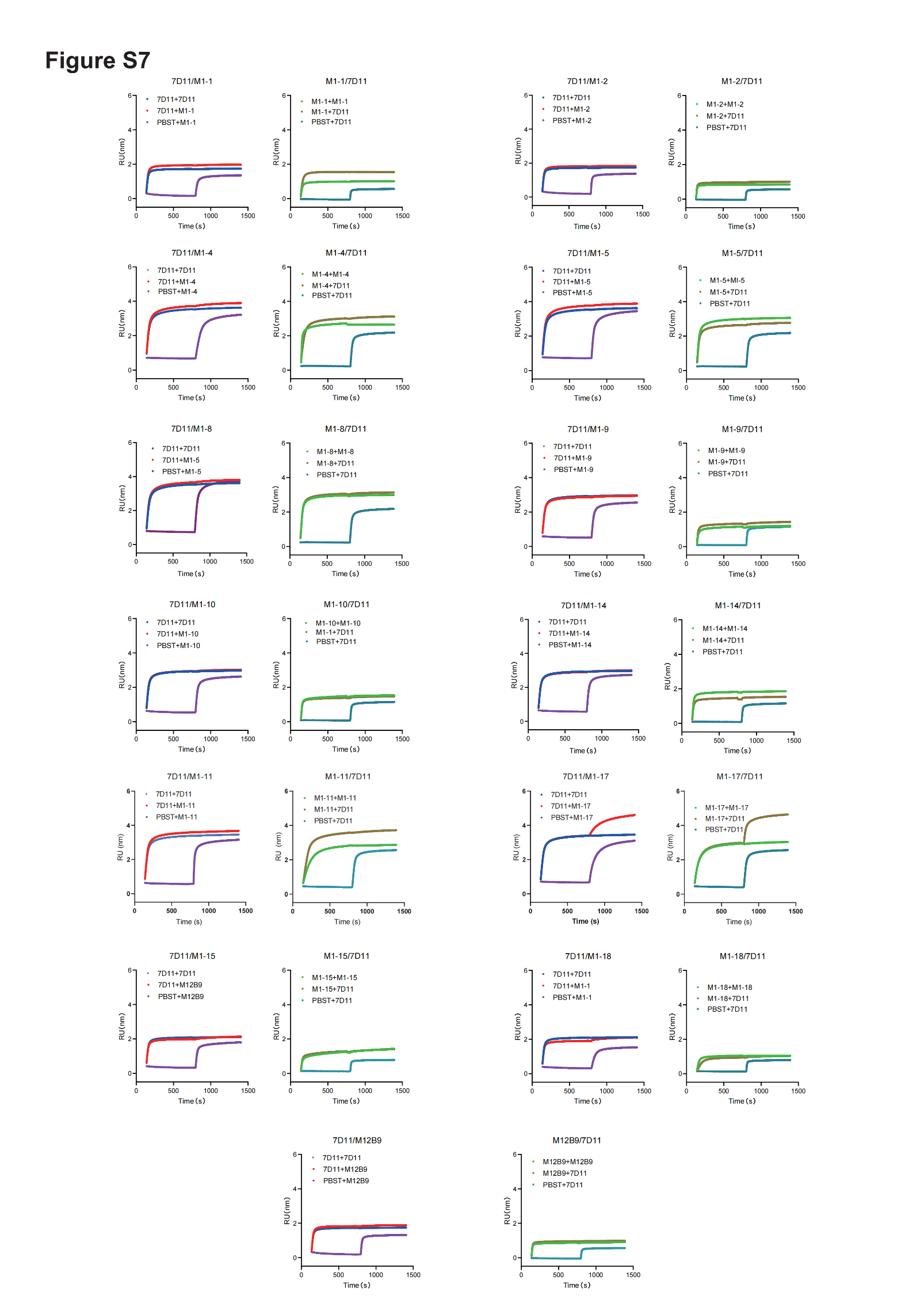
**

**Supplementary Figure 7. Competition results of the top frequent neutralizing mAbs elicited by M1 vaccinations against reference antibody 7D11.** The BLI technique was used to identify whether the neutralizing mAbs elicited by M1 vaccinations competitively bind M1 with reference antibody 7D11. M12B9 is a positive control targeting the same epitope of 7D11 and M1-17 is a negative control.


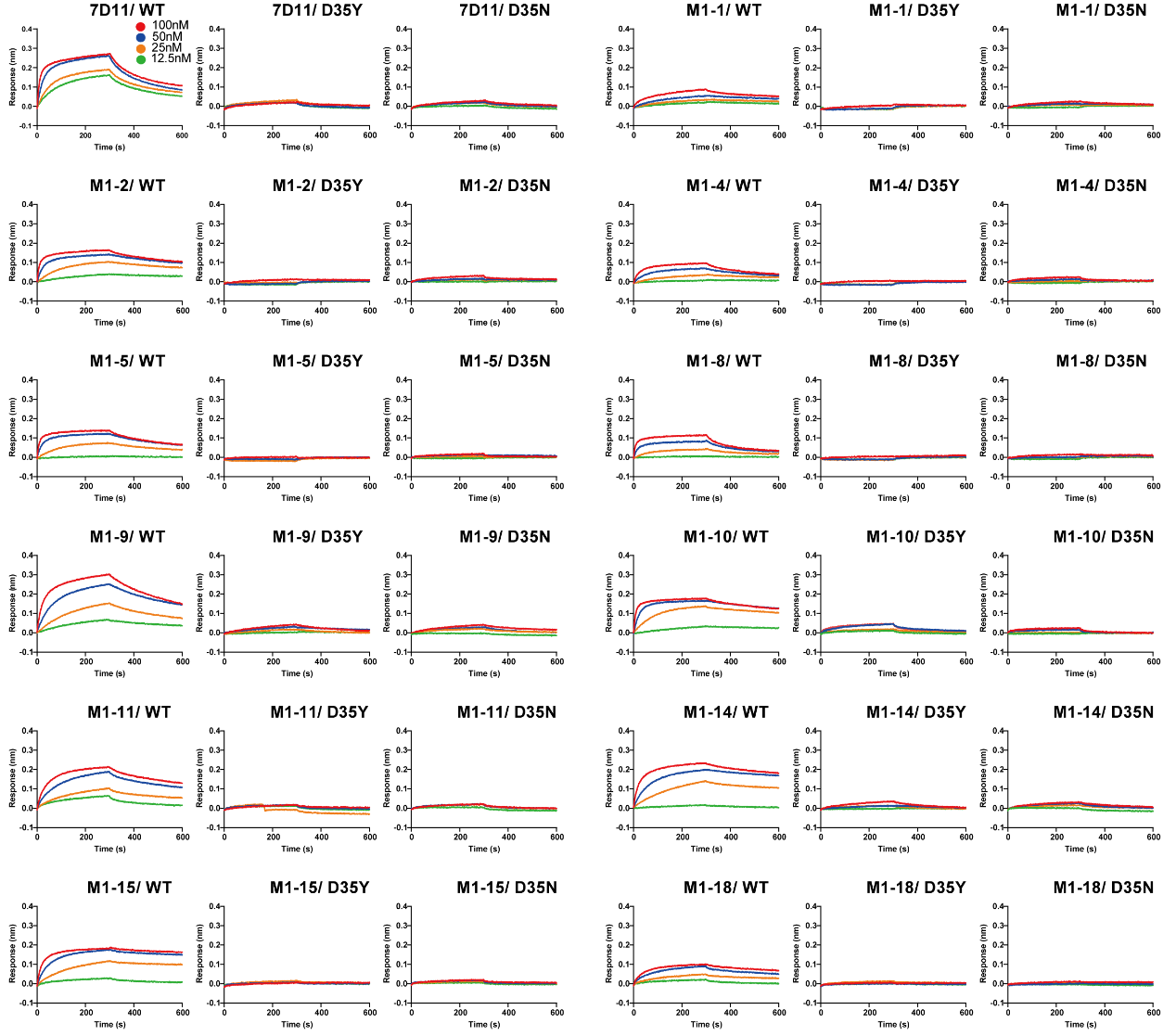


**Supplementary Figure 8. Binding results of the top frequent neutralizing mAbs with D35N or D35Y single substitutions of mpox M1 antigen.** BLI assays were done to determine if M1 antigen single substitutions cause neutralization escape. M1 wildtype antigen, D35N single substitution, and D35Y single substitution were used for detection with four concentrations. (curve red：100 nM, curve blue：50 nM, curve orange：25 nM, curve green：12.5 nM). 7D11 is a reported mAb as control and the other 11 antibodies are neutralizing mAbs elicited by M1 vaccinations.
